# Enhanced burn wound healing by controlled-release 3D ADMSC-derived exosome-loaded hyaluronan hydrogel

**DOI:** 10.1093/rb/rbae035

**Published:** 2024-03-26

**Authors:** Delong Zhu, Ying Hu, Xiangkai Kong, Yuansen Luo, Yi Zhang, Yu Wu, Jiameng Tan, Jianwei Chen, Tao Xu, Lei Zhu

**Affiliations:** Department of Dermatology & Plastic Surgery, The Third Affiliated Hospital of Sun Yat-Sen University, Guangzhou 510630, China; Department of Dermatology & Plastic Surgery, The Third Affiliated Hospital of Sun Yat-Sen University, Guangzhou 510630, China; Department of Dermatology & Plastic Surgery, The Third Affiliated Hospital of Sun Yat-Sen University, Guangzhou 510630, China; Department of the Second Plastic Surgery, The First People’s Hospital of Foshan, Foshan 528000, China; Department of Research and Development, Huaqing Zhimei (Shenzhen) Biotechnology Co., Ltd, Shenzhen 518107, People’s Republic of China; Department of Dermatology & Plastic Surgery, The Third Affiliated Hospital of Sun Yat-Sen University, Guangzhou 510630, China; Department of Dermatology & Plastic Surgery, The Third Affiliated Hospital of Sun Yat-Sen University, Guangzhou 510630, China; Center for Bio-Intelligent Manufacturing and Living Matter Bioprinting, Research Institute of Tsinghua University in Shenzhen, Tsinghua University, Shenzhen 518057, People’s Republic of China; Center for Bio-Intelligent Manufacturing and Living Matter Bioprinting, Research Institute of Tsinghua University in Shenzhen, Tsinghua University, Shenzhen 518057, People’s Republic of China; Tsinghua Shenzhen International Graduate School, Tsinghua University, Shenzhen 518055, People’s Republic of China; Department of Dermatology & Plastic Surgery, The Third Affiliated Hospital of Sun Yat-Sen University, Guangzhou 510630, China

**Keywords:** adipose-derived mesenchymal stem cells, exosomes, 3D microfiber culture, hyaluronic acid hydrogel, burn, wound healing

## Abstract

Adipose mesenchymal stem cell (ADMSC)-derived exosomes (ADMSC-Exos) have shown great potential in regenerative medicine and been evidenced benefiting wound repair such as burns. However, the low yield, easy loss after direct coating, and no suitable loading system to improve their availability and efficacy hinder their clinical application for wound healing. And few studies focused on the comparison of biological functions between exosomes derived from different culture techniques, especially in exosome-releasing hydrogel system. Therefore, we designed a high-performance exosome controllable releasing hydrogel system for burn wound healing, namely loading 3D-printed microfiber culture-derived exosomes in a highly biocompatible hyaluronic acid (HA). In this project, we compared the biological functions *in vitro* and in a burn model among exosomes derived from the conventional two-dimensional (2D) plate culture (2D-Exos), microcarrier culture (2.5D-Exos), and 3D-printed microfiber culture (3D-Exos). Results showed that compared with 2D-Exos and 2.5D-Exos, 3D-Exos promoted HACATs and HUVECs cell proliferation and migration more significantly. Additionally, 3D-Exos had stronger angiogenesis-promoting effects in tube formation of (HUVECs) cells. Moreover, we found HA-loaded 3D-Exos showed better burn wound healing promotion compared to 2D-Exos and 2.5D-Exos, including accelerated burn wound healing rate and better collagen remodeling. The study findings reveal that the HA-loaded, controllable-release 3D-Exos repair system distinctly augments therapeutic efficacy in terms of wound healing, while concurrently introducing a facile application approach. This system markedly bolsters the exosomal loading efficiency, provides a robust protective milieu, and potentiates the inherent biological functionalities of the exosomes. Our findings provide a rationale for more efficient utilization of high-quality and high-yield 3D exosomes in the future, and a novel strategy for healing severe burns.

## Graphical abstract

**Figure:**
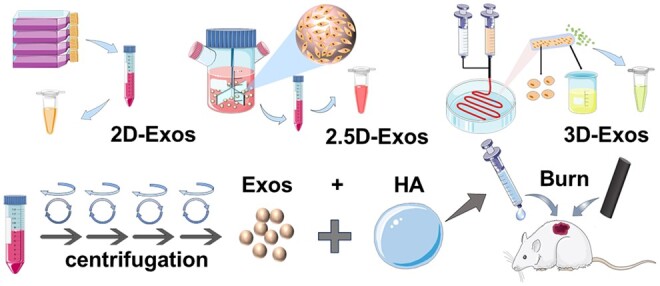

## Introduction

Burn trauma stands as the fourth predominant form of global injury, succeeded only by vehicular accidents, falls, and interpersonal violence [1–3]. Data from the World Health Organization (WHO) reveal that an estimated 11 million individuals necessitate medical intervention annually for burn injuries [4], contributing to a staggering 265 000 fatalities each year [5, 6]. These injuries are fraught with complex pathophysiological consequences, including immunological and inflammatory reactions [7, 8], metabolic alterations, and systemic shock, culminating in potential multiple organ failure [9]. Beyond the physiological ramifications, burn injuries exert a profound impact on psychological well-being and overall quality of life [10]. The current gold-standard for treating severe or expansive burn wounds is early excision followed by autologous skin grafting [6]. Nonetheless, the paucity of suitable autologous skin remains a formidable hurdle in regenerative outcomes [11, 12]. Therefore, an efficient treatment for severe burn wounds is urgently needed.

Exosomes, renowned for their therapeutic potential as cell-secreted extracellular vesicles (EVs) [13], have garnered colossal interest due to their roles in intercellular communication and wound healing facilitation. Stem cell-derived exosomes mirror the biological effects of their source cells [14], Besides lower immunogenicity and absent tumorigenicity, showcasing superior efficacy in wound healing promotion [15, 16]. They expedite wound healing by fostering angiogenesis [17], cell proliferation, and striking a balance in inflammatory responses [18]. Particularly, exosomes, when genetically modified or amalgamated with materials [19], demonstrate enhanced therapeutic properties including active ingredient enrichment, targeted delivery, and overcoming physiological barriers to penetration [20]—traits not present in traditional singular products[21, 22]. Moreover, exosomes serve diagnostic [23] and therapeutic purposes in wound-related applications, such as complex wound repair [24], graft success enhancement [25], treatment of related complications, and acting as diagnostic biomarkers [26]. Yet, the severely limited yield and propensity for loss of exosomes curtail their full potential realization. The yield limitation profoundly restricts their application, with loss and functionality further hindering the effectiveness of their use.

**Figure 1.**
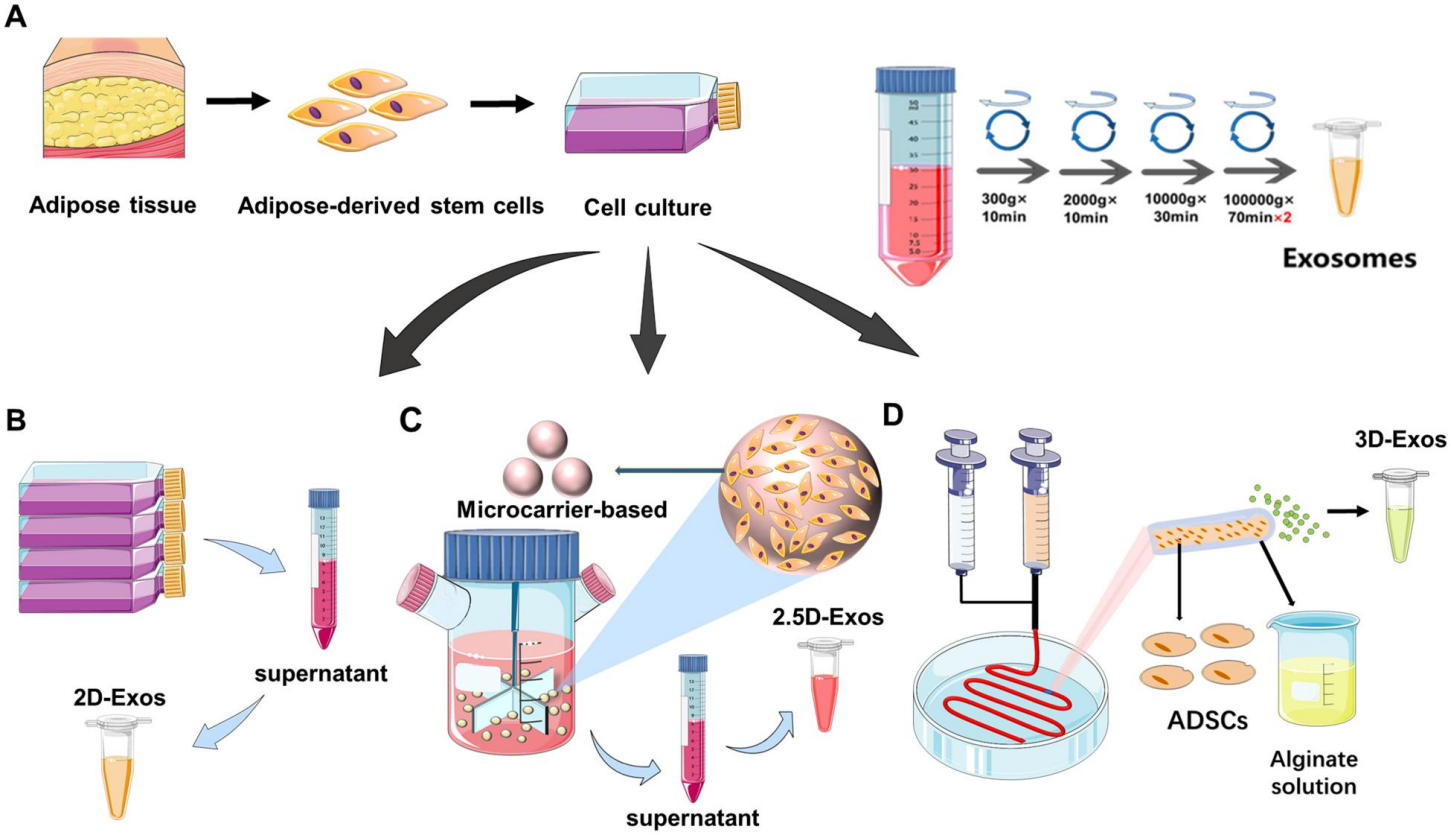
Schematic diagram of different processes to culture ADMSCs for exosomes extraction.

In recent years, mesenchymal stem cell (MSC)-derived EVs have emerged as promising candidates in regenerative medicine, although their broad adoption is stymied by challenges in scalable, high-yield production. Factors such as culture environment, isolation protocols, and purification methodologies play pivotal roles [27–29]. Recent empirical studies have illustrated that three-dimensional (3D) culture systems surpass traditional two-dimensional (2D) platforms in EV yield. Yet, the term ‘3D culture’ encapsulates a plethora of methodologies, ranging from microcarrier-based bioreactors to 3D coaxial bioprinting systems. Notably, microcarrier cultures in bioreactors [30], dubbed 2.5D-Exos, have showcased facile scalability for proliferating both primary and pluripotent stem cells, with significantly augmented surface areas of these microcarriers facilitating elevated cellular yields within a clinically pertinent timeframe (Figure 1C) [31–33]. This paves the way for an ‘on-demand’ off-the-shelf approach. In contrast, in 3D self-assembled microfiber cultures [26], termed 3D-Exos, a substantial quantity of adipose-derived mesenchymal stem cells (ADMSCs) (∼3 × 10^8^ total cells) are encapsulated within a meter-long hollow hydrogel-microfiber using coaxial bioprinting technology (Figure 1D). Within this 3D core-shell ultrafine fibrous biomimetic milieu, not only is there an augmentation in exosome yield, but there is also an enhanced expression of pluripotency markers (Oct4, Nanog, Sox2) in ADMSCs, while preserving their differentiation capabilities. This led us to delve into and compare these three distinct culture methodologies. The aforementioned developments, exemplified by microcarrier culture and 3D microfiber culture, primarily address the low yield of exosomes, elevating the production by 6- to 40-fold and 1000-fold, respectively. This lays a solid foundation for the clinical application of exosomes, albeit a paucity of clinical application precedents and unclear efficacy persist.

Although research has found that different cultivation methods can enhance the production of exosomes, a comprehensive comparison of their effectiveness in promoting the healing of burn wounds is still lacking. Additionally, exosomes face challenges such as rapid deactivation and low targeting efficiency when applied to wounds, urgently necessitating a novel exosomal delivery system to improve survival rates and targeting after application to wounds. Hyaluronic acid (HA), a ubiquitous glycosaminoglycan found in the extracellular matrix of all vertebrates [34], is lauded for its biological attributes including biocompatibility [35], biodegradability, non-immunogenicity [36], and non-toxicity, alongside being mechanically applicable [37]. The recent surge in interest has seen HA deployed in a myriad of applications from wound healing and tissue engineering to dentistry and targeted gene delivery [38, 39]. Exosomes [14], the microvesicular entities, have ascended as quintessential facilitators of cell–cell communication via paracrine pathways. Their integration into HA-based controlled-release systems heralds a promising avenue in wound repair strategies, showcasing potential as a vehicle [40, 41]. Effective amalgamation with exosomes could unveil new utilitarian pathways, however reports on its application in burn wound surfaces are scant.

In conclusion, a comprehensive system capable of combine high-efficiency exosome purification, process and retention during application, alongside high activity, remains elusive. This study innovatively proposes a holistic therapeutic strategy, intertwining high-yield, high-quality exosome production systems with HA slow-release systems, orchestrating a wound repair system and providing a comprehensive reference for subsequent tissue regeneration. Additionally, we scrutinized the biological functions *in vitro* and in a burn model among exosomes derived from conventional 2D plate culture (2D-Exos), microcarrier culture (2.5D-Exos), and 3D-printed microfiber culture (3D-Exos). Our findings revealed that the 3D system significantly enhanced cell migration, proliferation, and angiogenesis *in vitro*, as well as regenerative capabilities in *in vivo* burn wound healing. Our findings illuminate the superior therapeutic potential of 3D-Exos, especially when deployed in conjunction with an HA hydrogel-based delivery system, laying the foundation for novel treatment paradigms in the management of severe burn injuries.

## Materials and methods

### Human ADMSCs culture and 2D conventional cell culture

Human adipose-derived stem cells were obtained from Sciencell™ Research Laboratories (Figure 1A) and cultured in a 150-mm cell culture dish (NEST) at 37°C and 5% CO_2_ with 25–30 ml of Dulbecco’s modified Eagle medium (DMEM) (Gibco) supplemented with 10% fetal bovine serum (FBS) (Gibco) and 1% penicillin–streptomycin (Gibco). Serum-free ADMSCs culture medium was used as conditioned medium for Exos collection (Figure 1B).

### 2.5D ADMSCs microsphere preparation method

ADMSCs cells are observed under the microscope to observe the cell morphology, and can be planted in good condition. After digesting the cells, take out the Bioreactor, add 2–10 ml microcarriers, 5–20 million cells and 50–100 ml MSC medium in the culture bottle, put the culture bottle on the magnetic stirrer, set the interval to start, 35 rpm, 10 min; 0 rpm, 1 h. When the ADMSCs in the culture bottle proliferate to 8–10 times, add all the microcarriers from the bottle to the large culture bottle (Figure 1C). transferred to the large culture flask, added appropriate amount of new microcarriers, expanded the culture, and subsequently changed to serum-free medium for 2 days [42, 43].

### 3D ADMSCs microfiber fabrication and culture

The setup for coaxial cell microfiber extrusion is shown in (Figure 1D). Materials and cells were extruded from a double-layer coaxial needle, with the inner channel of ∼0.38 mm diameter containing a cell suspension at 3 × 10^7^ cells ml^−1^ and the outer channel of ∼1.1 mm diameter containing 1.5% sodium alginate solution (Aladdin, China). The extrusion rates were 3–10 and 15–30 ml/h, respectively. The coaxial needle was fixed by an iron base, with its lower end inserted into a crosslinking pool containing 3% CaCl_2_ solution. The alginate shell flow rapidly gelled in the CaCl_2_ solution to form a hydrogel envelope around the cell-containing core. After ∼10 min, the microfibers were transferred to new 150-mm culture dishes with 0.9% NaCl (Kelong, China) solution for 5 min and 25–30 ml MSC chemically defined medium (Yocon, China) at 37°C, 5% CO_2_. The extrusion power was provided by a syringe pump, which could adjust the inner and outer flow rates as needed [26] (according to literature, the recommended reference values for shell extrusion rate were 15–30 ml/h, and for core extrusion rate were 3–10 ml/h).

### Conditioned medium collection and preparation

For 2D culture, approximately 1.5 × 10^6^ ADMSCs were seeded into the same culture dish, cultured until 60–80% confluent, washed with phosphate-buffered saline (PBS) (Gibco, USA) 2 times and 3 times for 5 min each, to remove serum proteins and EVs, and the medium was changed to serum-free. After culturing for 48 h, conditioned chemically defined medium was collected. For 2.5D and 3D cultures, serum-free conditioned medium was directly collected every 24 h or 48 h according to optimization. All conditioned media were stored at 4°C or −80°C before Exos isolation.

### Isolation and identification of Exos

Exosomes were isolated from the conditioned medium of ADMSCs cultured under 2D, 2.5D, and 3D conditions by standard ultracentrifugation (UC) methods with slight modifications, referring to Ref. [44]. Briefly, to eliminate fragments and dead cells, we first pelleted intact cells at 300 × g for 10 min, then dead cells at 2000 × g for 30 min. Cell- and fragment-free conditioned medium was purified by pelleting at 10 000 × g for 30 min. The supernatant was filtered through a 0.22-μm filter (SLGV033RS, Millipore, USA) to remove vesicles or protein aggregates. Then the supernatant was spun at 100 000 × g for 70 min. The resulting pellet was washed with PBS, transferred to a new tube, and spun at 100 000 × g for 70 min. The pellet was resuspended for subsequent experiments. All spins were performed using an Optima MAX-XP ultracentrifuge (Beckman Coulter, USA) at 4°C.

### Exosomes were characterized for morphology, size distribution, and surface markers by TEM, NTA, and western blot

The morphology and ultrastructure of exosomes were analyzed by transmission electron microscopy (TEM) (JEOL, Tokyo, Japan). Exosome yield from 1 × 10^7^ cells was quantified by microBCA protein assay kit (CWBio, Beijing, China) and nanoparticle tracking analysis (NTA) according to manufacturer’s instructions. Levels of exosomal surface proteins CD81 and TSG101 were determined by western blot analysis.

### Tubule formation assay

Tube formation assay was performed to assess the angiogenic properties of EVs according to published literature [45]. Fifty microliters Matrigel containing 50% DMEM was dropped into one well of a 96-well plate, then placed in a 37°C incubator for 30-min gelation. HUVECs were cultured in FBS-free medium and starved for 12 h before seeding on the Matrigel surface. HUVECs (2 × 10^4^ cells/well) in 1% FBS DMEM with or without 2D-(5 × 10^8^ particles/ml), 2.5D-(5 × 10^8^ particles/ml) EVs, 3D-(5 × 10^8^ particles/ml) EVs were plated into the 96-well plate (Corning, USA) with Matrigel coating. After incubating for 2 h, images were taken under a microscope and analyzed using ImageJ ‘Angiogenesis Analyzer’ plugin. The number of meshes, nodes, total branch length, and total nodes were compared.

### Transwell assay

HUVECs were cultured in DMEM supplemented with 10% FBS under a controlled atmosphere of 5% CO_2_ at 37°C. A density of 1×10^4^ HUVECs was seeded into the upper chamber of a Transwell insert (Corning, 3422), while 600 μl of either fresh medium or exosome-enriched culture supernatant containing 10% FBS was added to the lower chamber [46]. Cells were subjected to a 12-h treatment with culture supernatants from either 2D-Exos, 2.5D-Exos, or 3D-Exos; a parallel cohort devoid of supernatant served as the control group. For migration assays, cells were incubated for an additional 12 h. Subsequently, the migrating cells that had traversed the basolateral membrane were fixed with 4% paraformaldehyde and stained with 0.1% crystal violet. Cellular invasion was visualized and documented using an optical microscope (Mshot, China).

### *In vitro* proliferation

The CCK-8 assay was used to determine the effects of different Exos on the proliferation of human immortalized keratinocyte (HaCaTs) cells and human umbilical vein endothelial cells (HUVECs). HaCaTs and HUVECs were seeded into 96-well plates at 2.5 × 10^3^ cells/well for 12 h, respectively. Cells were divided into 4 groups and treated with or without 2D-(5 × 10^8^ particles/ml), 2.5D-(5 × 10^8^ particles/ml) EVs, 3D-(5 × 10^8^ particles/ml) ADMSC-Exos. Five wells were set up for each group. CCK-8 reagent of 10 μl was added into 96-well plates at 12, 24, 48 and 72 h. After incubating in 37°C incubator away from light for 2 h, the absorbance at 450 nm was measured by a microplate reader to compare cell proliferation in each group.

### Migration assay

Scratch assay was performed to assess the effects of different Exos on the migration of HaCaT cells and HUVECs. HaCaTs and HUVECs were plated in 6-well plates at 5 × 10^5^ cells/well. When cell confluence reached 90%, cells were washed with PBS for 3 times after removing the medium and starved with FBS-free medium. Then a sterile 200 μl pipette tip was used to scratch the confluent cell monolayer. ADMSC-Exos of different groups at 5 × 10^8^ particles/ml were added into the wells. Images were taken at 0, 12, 24 and 48 h after the monolayer was scratched. The migrated area was quantified using ImageJ software: Migration area (%)=(A0−An)/A0 × 100%, where A0 is the initial wound area (*t* = 0 h) and An is the remaining wound area at the time of measurement (*t* = *n* h).

### Ethics statement

All procedures involving animals were conducted in strict adherence to the American Animal Protection Legislation, reflecting our commitment to the ethical and humane treatment of animals. The study protocols received formal approval from the Institutional Animal Care and Use Committee of The Third Affiliated Hospital of Sun Yat-Sen University (permission number: 02-180-01), ensuring that our research practices met the highest ethical standards established for animal care and use in scientific inquiry.

### *In vivo* burn wound healing

All animal experiments were carried out under the guidance of the Medical Ethics Committee of the Third Affiliated Hospital of Sun Yat-Sen University. Burn wound models were created on 32 Balb/c mice (male, 8 weeks old) according to established models [47, 48]. Mice were anesthetized by intraperitoneal injection of 50 mg/kg pentobarbital sodium. After shaving the back, full-thickness burn wounds were made using an iron rod (diameter: 14 mm, 100°C, 30 s). Forty-eight hours later, necrotic skin was removed and silicone splints were adhered around wounds [49]. Mice were randomly divided into 5 groups according to different postoperative treatments: Control, HA, HA + 2D-Exos, HA + 2.5D-Exos and HA + 3D-Exos. HA hydrogels were applied twice on the wounds, each time with 5 × 10^8^ particles of Exos, on Days 0 and 3. Then the wounds were covered with 3M Tegaderm and gauze. Images of the wounds were taken to analyze wound area using ImageJ software at predetermined time points (7, 14 and 21 days). The percentage of wound area was calculated as:

Wound area (%)=residual size/original size×100%.

On Day 14, tissues around wounds were dissected for further experiments. Re-epithelialization and collagen deposition were confirmed by hematoxylin & eosin (H&E) and Masson staining. To analyze cell proliferation and angiogenesis in wound areas, immunofluorescent staining and immunohistochemical staining were performed.

### Histochemistry and immunohistochemistry

For histochemical and immunohistochemical analyses, wound tissues were fixed in 4% paraformaldehyde solution and dehydrated in ethanol. Tissues were then embedded in paraffin and sectioned perpendicularly to the wound surface. Sections were stained with H&E and Masson’s trichrome (MT) for tissue samples according to routine protocols. H&E staining was used to examine new tissue formation and infiltrating cells. MT staining was used to detect collagen deposition. Images were captured by light microscopy.

To assess angiogenesis in different groups, immunohistochemical staining for CD31 was performed. Briefly, sodium citrate was used for antigen retrieval of tissues for 15 min. Tissues were then blocked with 3% bovine serum albumin for 30 min and incubated with anti-CD31 (1:100, Abcam) overnight at 4°C. The next day, horseradish peroxidase-conjugated secondary antibody was added for 50 min. Then cells were counterstained with hematoxylin. Finally, sections were imaged by light microscopy and analyzed using ImageJ.

### Exosome release kinetics

To examine the release of exosomes in HA + 2D-Exos, HA + 2.5D-Exos and HA + 3D-Exos groups, exosomes were mixed with HA at 5×10^7^/μl and incubated in 200 μl PBS at 37°C. At each timepoint, 20 μl PBS was collected and replenished with equal fresh PBS. The amount of exosomes was calculated by microBCA protein assay kit (Pierce™) for 48 h.

### Statistical analysis

Data are expressed as the mean±SD of at least three independent experiments (*n* ≥ 3). Statistical analysis was performed using independent sample *t*-tests for comparisons between two groups or one-way ANOVA for comparisons between multiple groups. *P* < 0.05 was considered statistically significant.

## Results

### Characterization of ADMSC-Exos

**Figure 2.**
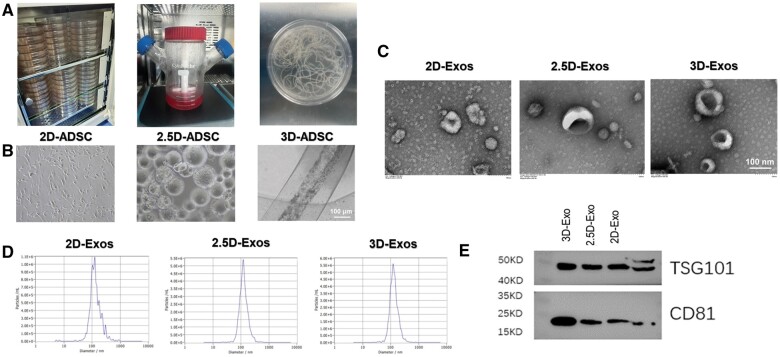
Characterization of 2D-Exos, 2.5D-Exos and 3D-Exos. (**A**) The photograph of 2D culture, 2.5D culture and 3D culture. (**B**) Bright-field images of 3D culture, 2.5D culture and 2D culture ADMSCs (scale bar = 100 μm). (**C**) Transmission electron microscopy images showing the morphology of 2D-Exos, 2.5D-Exo and 3D-Exo (scale bar=100 nm). (**D**) Particle size and number of 2D-Exos, 2.5D-Exos and 3D-Exos were analyzed using NTA. (**E**) Representative protein blot images showing the expression of exosome markers (CD81 and TSG101) in 2D-Exos, 2.5D-Exos and 3D-Exos.

Multicellular spheroids were formed by ADMSCs in a 3D coaxial scaffold culture system [26], contrasting with the spindle-like morphology of ADMSCs in 2.5D microcarrier culture and 2D conventional planar culture. These results indicate the successful construction of an alternative cell culture model (see Figure 2A and B). Exosomes were extracted from the supernatants of ADMSCs grown on coaxial scaffolds (3D-Exo), microsphere cultures (2.5D-Exo), and 2D planar cultures (2D-Exo) using standard UC methods. As observed through TEM, 2D, 2.5D, and 3D Exos were nano-sized vesicles that possessed a bilayer membrane measuring approximately 120 nm [44], aligning with exosomes’ reported size distribution (Figure 2C) [13, 42]. NTA indicated that the diameters of 2D, 2.5D and 3D-Exos ranged from 50 to 200 nm according to Figure 2D. Positive expression of exosome surface proteins CD81 and TSG101 was shown through protein blotting analysis in accordance with Figure 2E [50]. In conclusion, the results demonstrate the successful isolation of multiple exosomes cultured through various processes.

### Promoting keratinocyte and endothelial cell migration and proliferation *in vitro*

**Figure 3.**
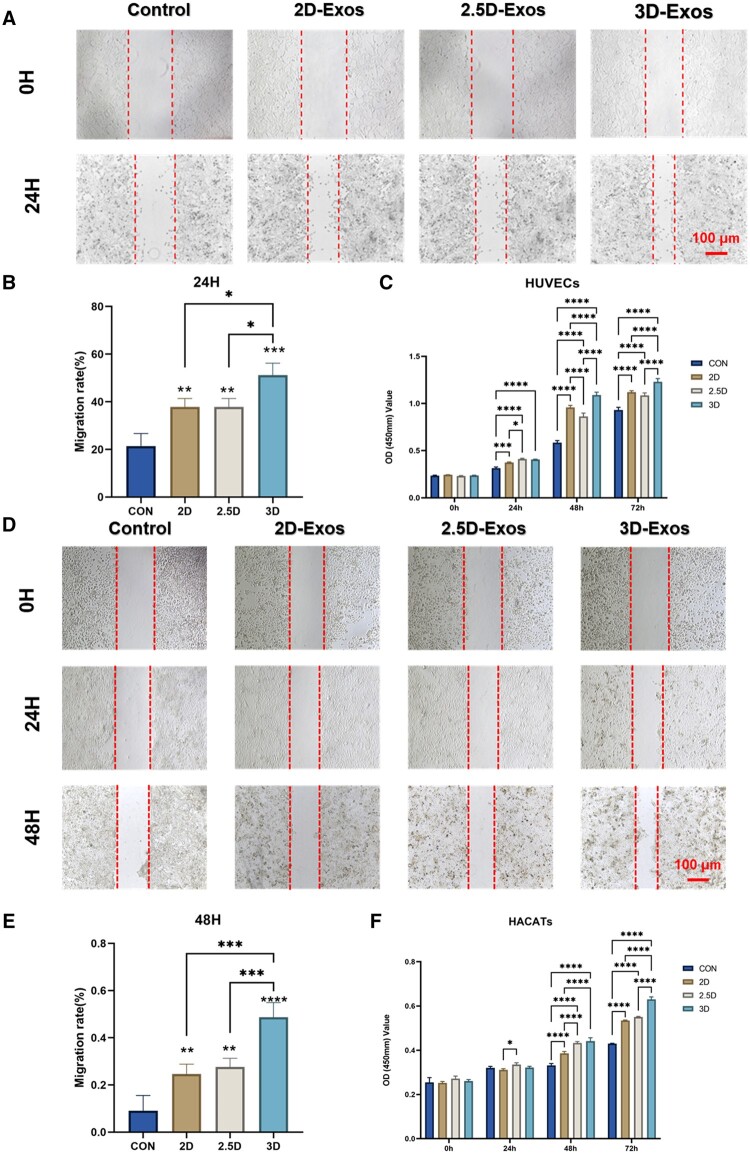
Effects of different ADMSCs exosomes on proliferation and migration of HUVEC versus HACATs. (**A, D**) Scratch assay of HUVECs in the indicated treatment groups (scale bar: 100 μm). (**B, E**) Scratch healing in the indicated groups was quantified, revealing enhanced migration after 3D-Exos treatment. (**C, F**) HUVECs in the indicated treatment groups were compared with the HACAT cells for indicators detected by CCK-8 assay after 24, 48 and 72 h of culture (*P<0.05; **P<0.01; ***P<0.001; and ****P<0.0001).

The traditional skin wound healing process involves four classic stages: hemostasis, inflammation, proliferation, and maturation [51]. A meta-analysis has shown that exosome treatment plays biological roles in wound healing, mainly by accelerating skin cell proliferation, re-epithelialization [52, 53], angiogenesis [54, 55], and anti-inflammatory effects [20]. To better elucidate the effects of Exos treatment that could accelerate burn wound healing, we evaluated the impacts of these exosomes on wound healing related cell types (endothelial cells and keratinocytes). CCK-8 assay was applied to determine the effects of 2D, 2.5D and 3D-Exos on the proliferation of these two cell types. We used HaCaT cells, an immortalized mature human keratinocyte cell line, and human umbilical vein endothelial cells (HUVECs) in this study. As shown in Figure 3A and B, these different exosomes promoted proliferation of HaCaTs or HUVECs at 5 × 10^8^/ml concentration *in vitro*. In the *in vivo* wound healing assay, the wound closure rate was significantly increased in the 2D, 2.5D and 3D-Exos treatment groups compared to PBS control, with the 3D coaxial cultured exosome group showing the most significant effect.

Data derived from CCK-8 cell proliferation and scratch healing assays demonstrated consistency. Based on this analysis, we concluded that exosomes produced via 3D-printed microfiber culture markedly enhance the migration and proliferation capabilities of endothelial cells and keratinocytes. It is particularly noteworthy that, although exosomes from different cultivation techniques all showed this promotive effect, those derived from 3D-printed microfiber technology exhibited the most significant enhancement in cellular functions.

In the event of burn injuries, the impacted site is subjected to a high reactive oxygen stress microenvironment. To address this, our study has been extended to include a new experimental group. This group focuses on applying ADMSC-Exosomes, synthesized via multiple techniques, to HaCaT cells treated with H_2_O_2_, thereby mimicking the oxidative stress environment typical of burn wounds. This enhancement of our research protocol is designed to deepen our understanding of the impact of ADMSC-Exosomes, prepared under varying conditions, in high oxidative stress settings. Such an investigation is crucial for advancing our knowledge of the therapeutic potential of ADMSC-Exosomes in the context of burn wound healing under oxidative stress.

The scratch wound healing assay results indicated an enhanced migratory capability of HaCaT cells cultured with ADSC-Exosomes, irrespective of the presence or absence of H_2_O_2_. However, the effect of 3D-Exos on the migration of HaCaT cells, which was impaired by H_2_O_2_, was particularly notable (as shown in Supplementary Figure S2A and B; *P < 0.05, **P < 0.01, ***P < 0.001). In Supplementary Figure S2C, we observed an increased optical density at 450 nm in the 3D-Exos group compared to the control group. This enhancement was also evident in the 3D-Exos+ H_2_O_2_, 2.5D-Exos+ H_2_O_2_, and 2D-Exos+ H_2_O_2_ groups in comparison to the H_2_O_2_ group. Notably, 3D-Exos significantly improved the proliferation of HaCaT cells that were impaired by H_2_O_2_.

These findings collectively suggest that ADMSC-Exosomes from different preparation methods facilitate cell proliferation and migration, with the 3D-Exos+ H_2_O_2_ group showing the most pronounced effect. This highlights the potential of 3D-Exos as a significant factor in skin wound healing, particularly in environments characterized by high reactive oxygen stress. This study provides valuable insights into the regenerative capabilities of ADMSC-Exosomes under oxidative stress conditions, reinforcing their therapeutic potential in wound healing applications.

### Different exosomes promote angiogenesis

**Figure 4.**
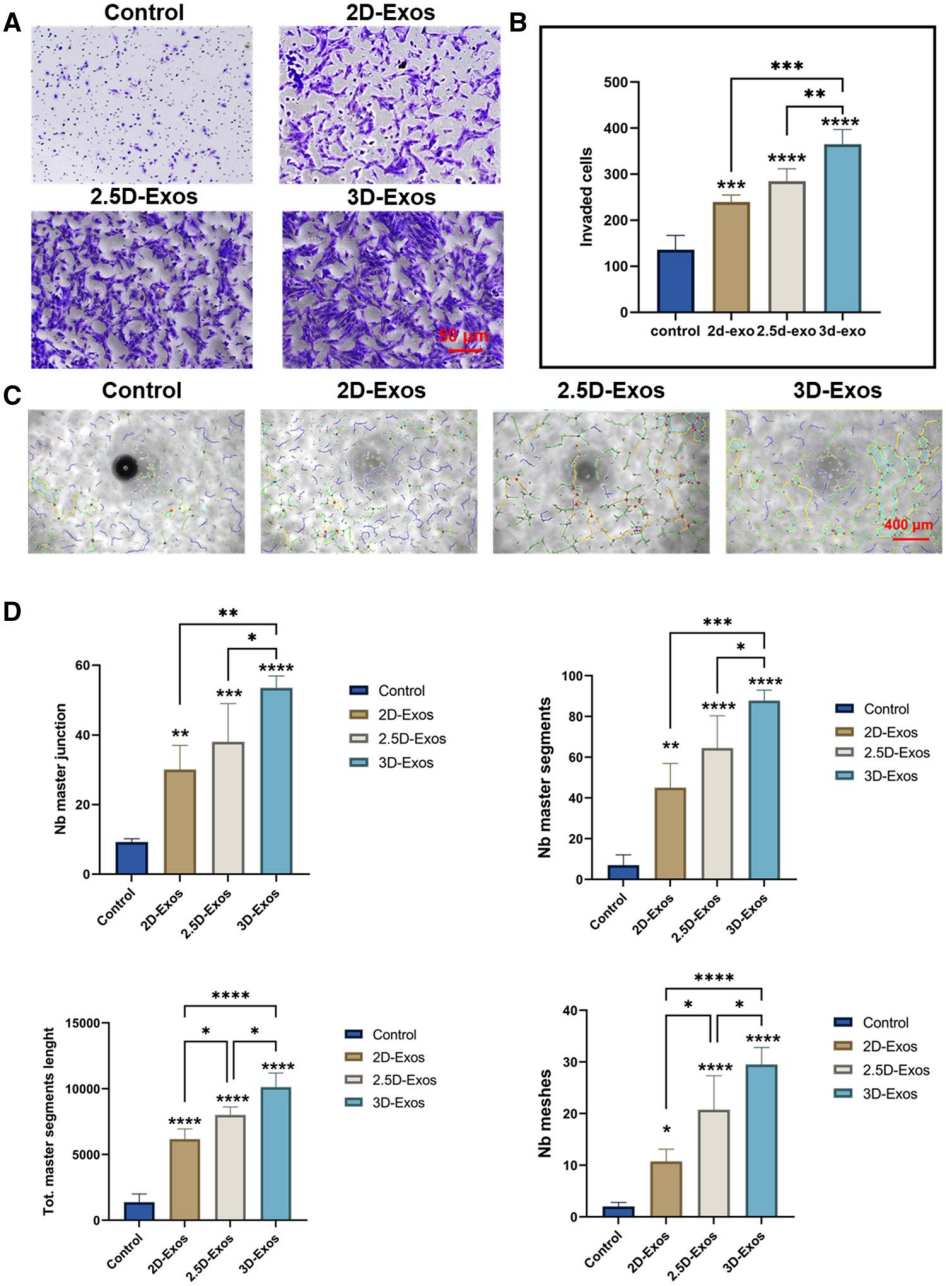
(**A**) Transwell migration assay of HUVECs in the indicated treatment groups. (**B**) Quantification of the number of migrating HUVECs in the Transwell assay reveals a greater migration capacity after 3D-Exos treatment than 2.5D-Exos, 2D-Exos and control (scale bar: 50 μm). (**C**) HUVECs in the indicated treatment groups were used in an *in vitro* tube formation assay. HUVECs cells were used for *in vitro* tube formation assay. (**D**) Quantification of the number of tubes per field of view, revealing that 3D-Exos treated HUVECs have a stronger tube formation capacity than 2.5D-Exos, 2D-Exos and control (scale bar: 400 μm). Error bars were calculated based on triplicate samples (*P <* *0.05; **P <* *0.01; ***P <* *0.001; and ****P <* *0.0001).

Angiogenesis is the biological process of new blood vessel formation [56], which not only involves the abilities of endothelial cell proliferation and migration, but is also closely related to their tube formation ability [45], as the newly formed vessels can deliver oxygen and nutrients to the wound site. To better evaluate the wound healing outcomes of different exosomes in burns, we seeded different exosome pretreated HUVECs on Transwell chambers or matrix gels. The total migrated cells and tube numbers were counted to assess the angiogenic ability of HUVECs. As shown in Figure 4A and B, compared with the control group, exosomes significantly enhanced the motility of HUVECs, and HUVECs treated with 3D exosomes had the strongest migratory ability, consistent with our scratch and proliferation experiments. In addition, better tube formation performance was observed in the 3D-Exos group versus 2D-Exos or 2.5D-Exos groups, characterized by higher tube numbers and more intact tube structures (Figure 4C and D). This not only confirmed previous studies that exosomes can promote angiogenesis and facilitate wound healing, but also indicated 3D-Exos exerted enhanced angiogenic abilities in the tube formation experiment.

### Characterization of HA hydrogel and controlled release of exosomes

In our quest to discover new methods of wound healing, we have focused on the diverse biological functions of hyaluronic acid (HA). Significantly, HA-based hydrogels, used as wound dressings, have emerged as vital contributors to the wound healing process. These hydrogels not only establish an anti-inflammatory milieu conducive to modulating immune responses but also demonstrate a profound capacity for controlled and sustained release of therapeutic agents, like exosomes.

**Figure 5.**
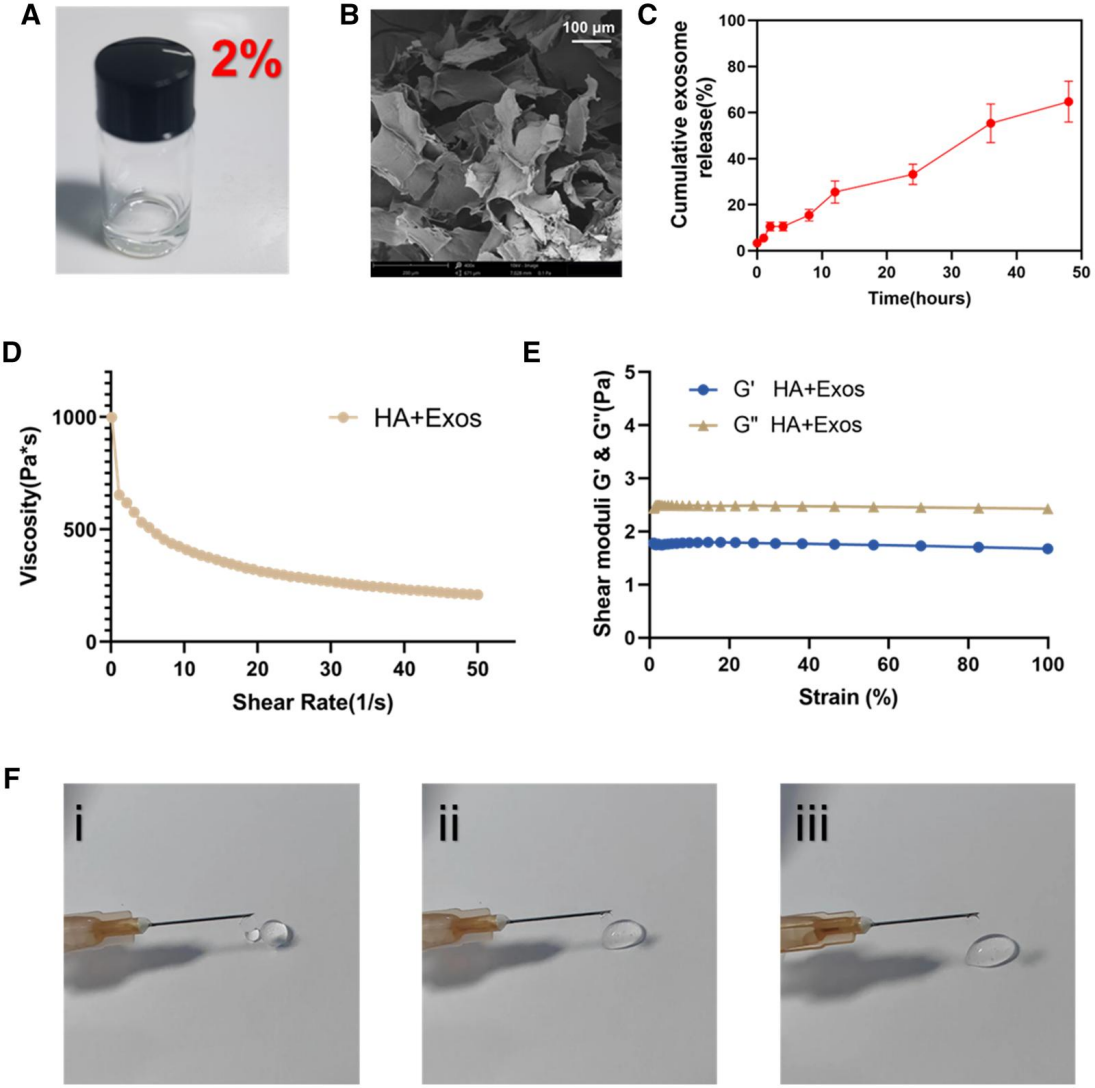
Preparation and characterization of HA + Exos. (**A**) The photographic image of a solution containing hyaluronic acid. (**B**) SEM image of HA (scale bar: 100 μm). (**C**) *In vitro* releasing curves of hyaluronic acid-exosomes. (**D**) Shear rate sweeps for HA + Exos. (**E**) Strain sweeps for HA + Exos. (**F**) A syringe could easily deliver the HA + Exos solution to produce droplets.

In an innovative approach, as depicted in Figure 5A, we have engineered a novel exosome controllable release hydrogel system specifically for the healing of burn wounds. This system incorporates exosomes derived from 3D-printed microfiber cultures into a biocompatible HA matrix. Following freeze-drying, illustrated in Figure 5B, the HA+Exos hydrogel exhibits a characteristic 3D porous morphology. Importantly, the addition of exosomes does not significantly alter the pore structure, indicating their effective integration within the hydrogel matrix. A key aspect of our research, shown in Figure 5C, involved examining the release kinetics of ADMSC-Exos from the HA+Exos hydrogel. Using the Exo microBCA protein assay kit, we observed a consistent release profile of the loaded exosomes. Impressively, the hydrogel facilitated a cumulative release of over 65.1% after 48 h. These findings are crucial, demonstrating that HA can be effectively utilized to achieve a controlled and sustained release of exosomes, an attribute that could be instrumental in advancing wound healing therapies. As illustrated in Figure 5D and E, the rheological properties of our HA+Exos hydrogel were elucidated through shear rate and strain sweeps. These properties not only ease the application of the HA+Exos but also amplify its sustained moisturizing effect. Moreover, the hydrogel’s optimized exosome release profile could potentially enhance the retention time of exosomes at the wound site. Figure 5F highlights the injectability of the HA, enabling surgeons to precisely adjust the hydrogel’s coverage area during surgical procedures. The commendable bioadhesive characteristics and controllability of our injectable hydrogel suggest that it could effectively form a protective barrier over injured skin.

To further corroborate the effective incorporation of exosomes into the HA matrix, we utilized ADMSC-derived exosomes, labeled with PKH26 red fluorescent dye, and integrated them into the HA. Intriguingly, our imaging techniques unveiled a uniform distribution of ADMSC-Exos within the HA matrix, as detailed in Supplementary Figure S1A. The subsequent investigations, depicted in Supplementary Figure S1B, entailed introducing these PKH26-labeled HA-Exos into the serum-free culture medium of HUVECs. Remarkably, post a 24-h incubation period, we observed significant accumulation of these labeled exosomes in the perinuclear area of the HUVECs. This observation is indicative of the successful release and efficient internalization of ADMSC-Exos by HUVECs.

### *In vivo* promotion of burn wound healing

**Figure 6.**
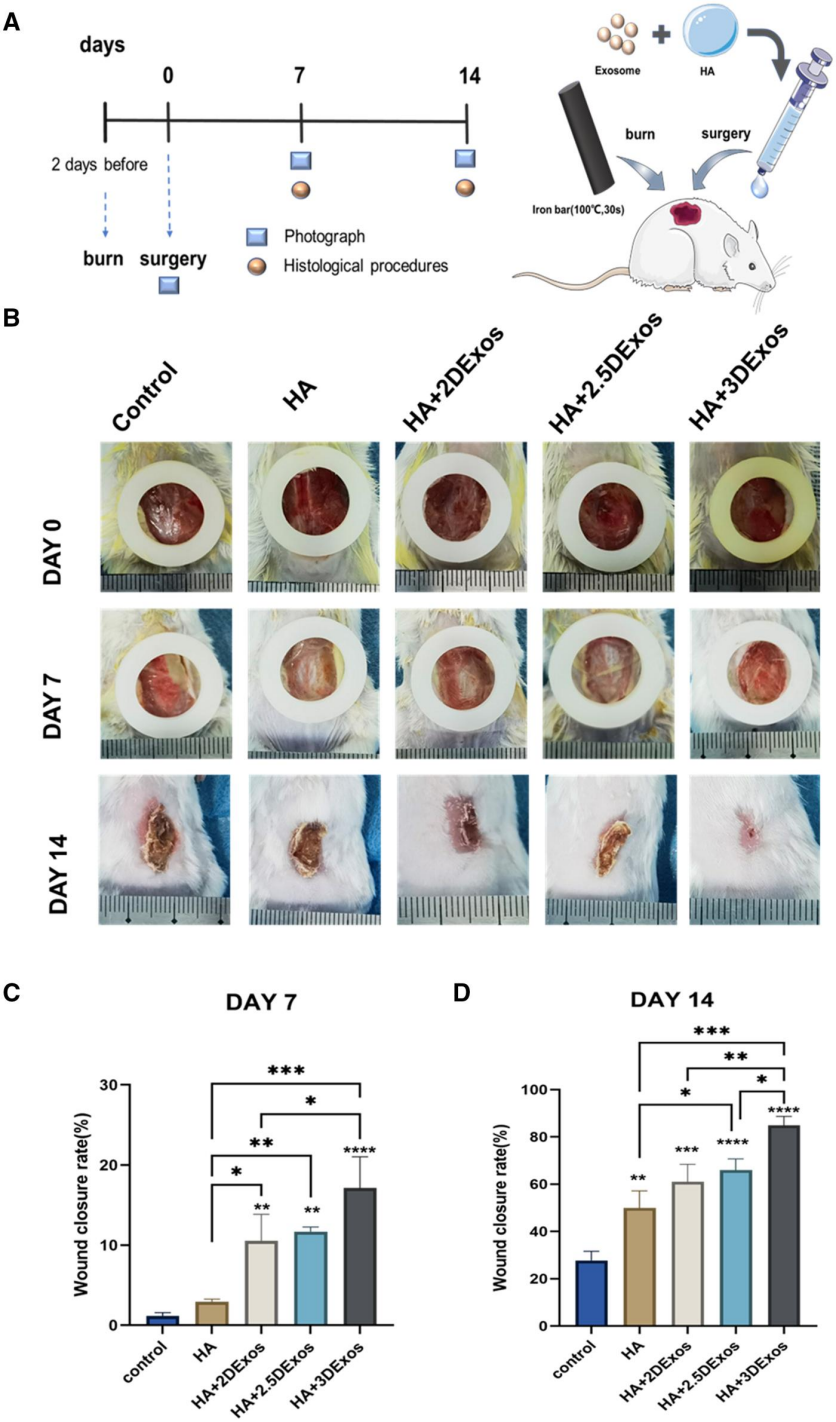
Hydrogel characterization with the effect of different ADMSC-exosomes on burn wounds. (**A**) Burn wounds were fabricated before 2 days and necrotic skin was removed on Day 0. Mice were randomly divided into five groups (*n* = 5 each) according to different treatments after surgery: control, HA, HA + 3D-Exos, HA + 2.5D-Exos and HA + 2D-Exos. Photographs of the wound area were performed on Days 0, 7 and 14, and mice were executed for IHC procedures on Days 7 and 14. (**B**) Representative images of the mouse burn wound healing process with different treatments over 14 days. (**C, D**) Quantification of wound area expressed as a percentage of initial wound size. Wound closure could be observed on Day 14, where the HA + 3D-Exos group showed the most significant wound healing effect (*P <* *0.05; **P <* *0.01; ***P <* *0.001; and ****P <* *0.0001).

To investigate the wound healing impact of diverse exosomes *in vivo*, we initially examined the finely tuned, exceptionally biocompatible HA using light and electron microscopy. Subsequently, the usage of the BCA Protein Assay Kit significantly decreased the exosome release rate [49]. In our study, a comprehensive skin burn model was established in BALB/c mice, as depicted in Figure 6A. Over time, we observed a progressive reduction in the wound area (Figure 6B). Notably, at 14 days post-implantation, HA supplemented with 3D-Exos exhibited significantly enhanced healing, achieving a closure rate of 84.91%±3.74%. This was superior to the rates observed with HA+2.5D-Exos (66.05%±5.34%), HA+2D-Exos (61.05%±7.20%), HA alone (49.94%±6.35%), and the control group (27.75%±3.46%), as detailed in Figures 6C and 6D. The results revealed that HA-3D-Exos presented greater wound-healing potential than the other groups. These findings confirm that exosomes promote wound healing, as previously noted. Additionally, the study demonstrated that 3D-Exos were more effective therapeutically in the skin burn model when compared to exosomes cultured by other means, supporting the earlier conclusions of the cellular experiments.

### Histological analysis of wound healing

**Figure 7.**
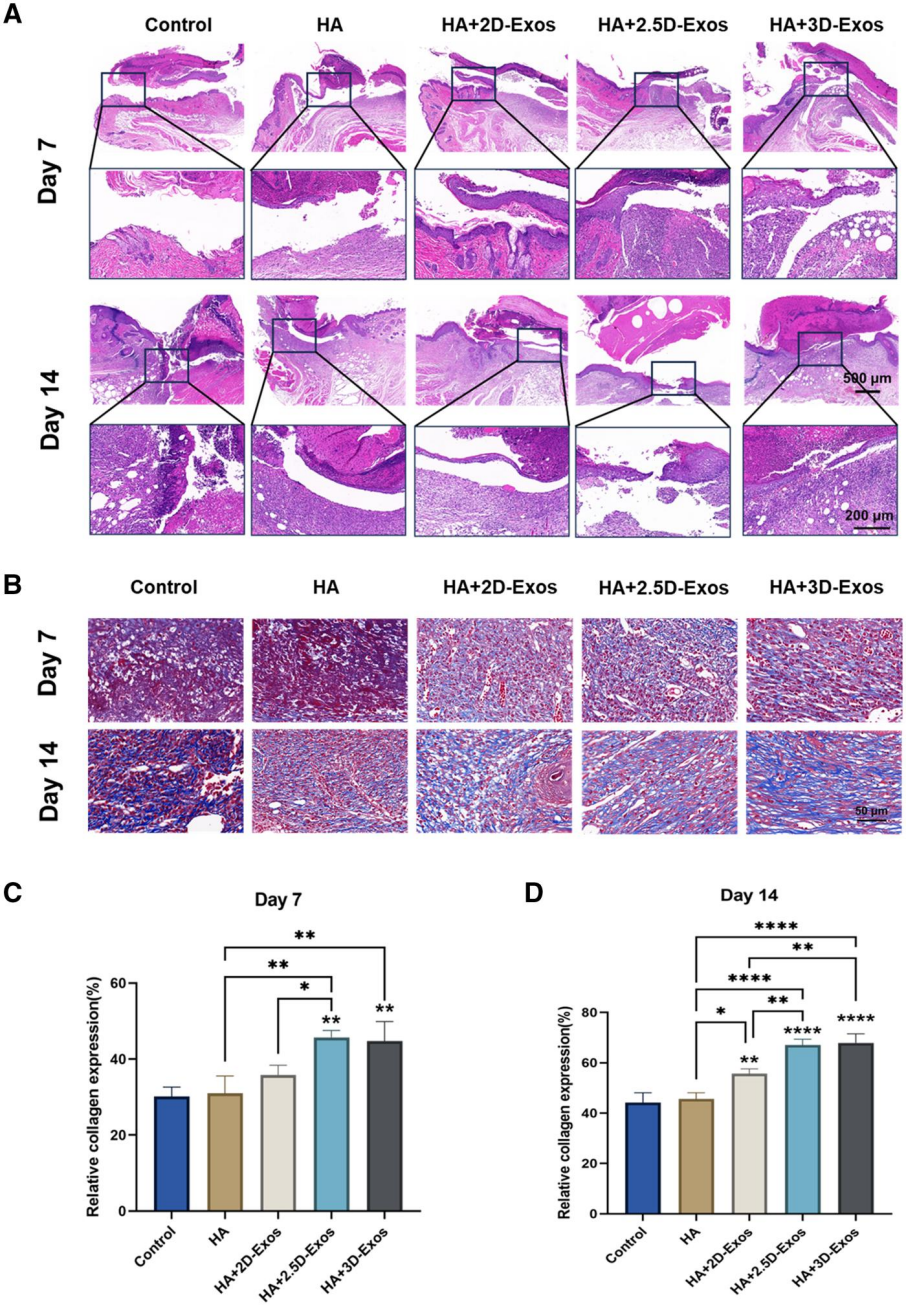
Histologic evaluation of wound healing (H&E staining) versus (Masson staining). (**A**) HE staining images of burn wound skin in mice with different treatments. At Days 7 and 14, the wounds treated with HA + 3D-Exos showed more complete skin structures (scale bar: 500 μm, 200 μm). (**B**) MT-stained images of burn wound skin in mice with different treatments. At Days 7 and 14, the expression of collagen in the wound tissue of the HA + 3D-Exos group was increased compared to the other groups (scale bar: 50 μm). (**C**, **D**) Quantitative comparison of collagen expression between different treatment groups at Days 7 and 14 (*P <* *0.05; **P <* *0.01; ***P <* *0.001; and ****P <* *0.0001).

As shown in Figure 7A, at Day 7, the wounds in the control group were still disrupted, while the exosome groups’ wounds, especially the HA + 3D-Exos, showed relatively regular structures to some extent. This indicates HA + 3D-Exos treatment accelerated re-epithelialization. Additionally, at Day 7, severe inflammatory cell infiltration was observed in the wound beds of control and HA groups, which was not displayed in the HA + 2D-Exos, HA + 2.5D-Exos and HA + 3D-Exos groups. This may be due to the anti-inflammatory effects of exosomes, which can modulate inflammatory cytokines (e.g. interleukin, monocytes, and neutrophils). Excessive/prolonged inflammation may inhibit wound healing [57], and anti-inflammatory dressings can accelerate wound healing. Moreover, re-epithelialization is an indispensable event in the wound healing process [58]. At 14 days, the re-epithelialization was even more pronounced; while the HA + 3D-Exos showed a more intact skin structure, other groups displayed irregular epithelial hyperplasia and hyperkeratosis. Specifically, treatment with 3D-Exos group produced more organized connective tissue and more complete skin structure relative to the other groups.

As shown in Figure 7B, the deposition of collagen in the wound beds of the HA + 3D-Exos group was denser and more continuous compared to other groups. Quantitative analysis (Figure 7C and D) of collagen deposition found the HA + 3D-Exos had significantly higher amounts of collagen versus Control, HA, HA + 2D-Exos and HA + 2.5D-Exos at both 7 and 14 days. Our histological results were consistent with previous studies, demonstrating exosomes can enhance collagen deposition in burn wounds. This may be because 3D-Exos are better at mimicking the microenvironment *in vivo* leading to better wound healing effects than the 2D-Exos and 2.5D-Exos groups.

### HA + 3D-Exos constructs enhanced angiogenesis *in vivo*

**Figure 8.**
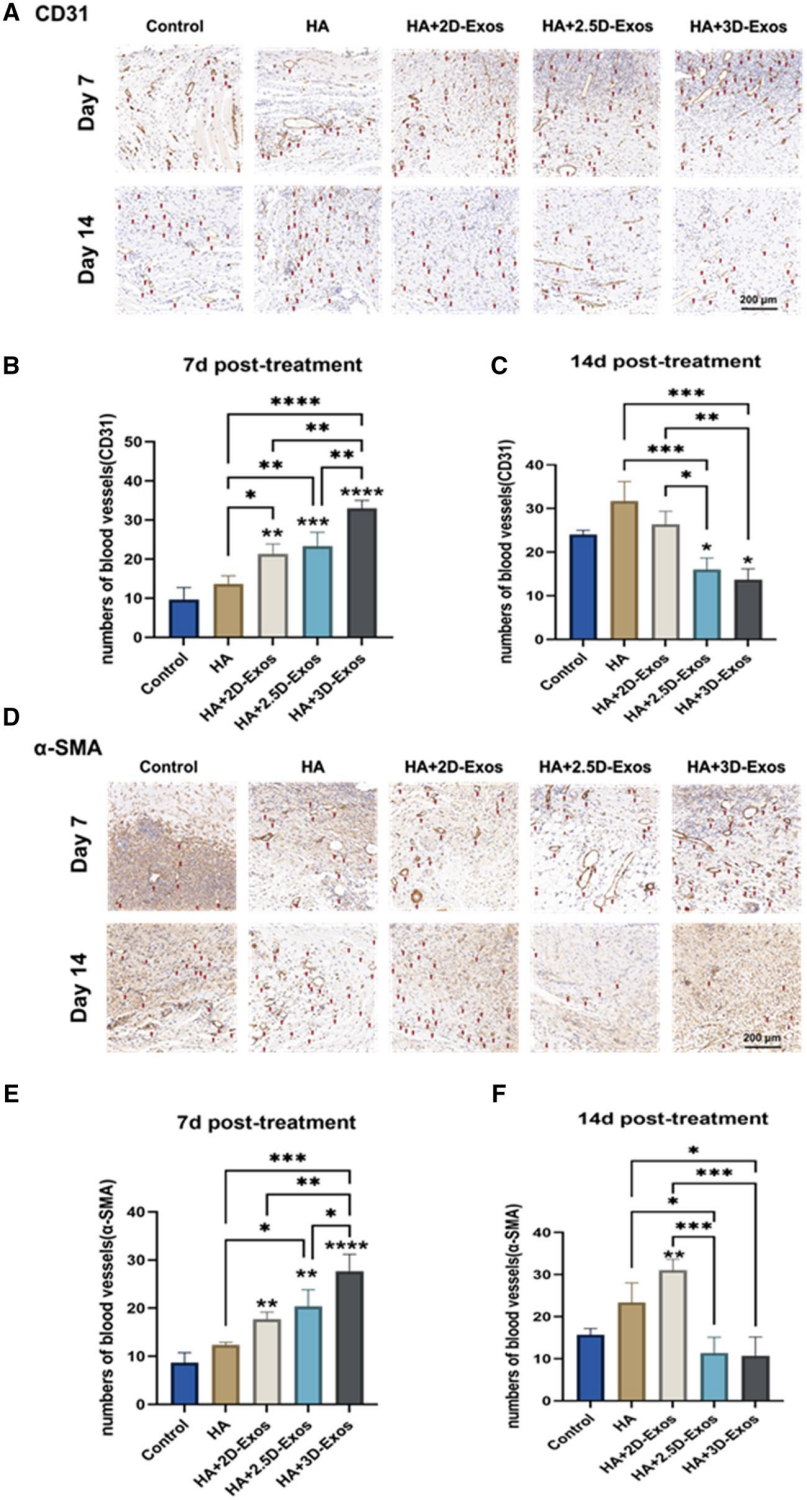
(**A**) CD31 staining of mouse burn wound skin at 7 and 14 days with different treatments. More mature blood vessels were observed in the HA + 3D-Exos group than in the control, HA, HA + 2.5D-Exos and HA + 2D-Exos groups (scale bar: 200 μm). (**B**, **C**) Quantitative comparison of CD31-positive (+) vessels at 7 and 14 days between different treatment groups. (**D**) α-SMA staining of mouse burn wound skin at 7 and 14 days with different treatments (scale bar: 200 μm). (**E**, **F**) Quantitative comparison of α-SMA-positive (+) vessels at 7 and 14 days between different treatment groups (*P <* *0.05; **P <* *0.01; ***P <* *0.001; and ****P <* *0.0001).

Angiogenesis is pivotal in wound repair, emerging as a fundamental target for effective wound healing strategies [54, 55]. Pro-angiogenic agents, including epidermal growth factor, fibroblast growth factor, and vascular endothelial growth factor, alongside angiogenic cells, have been utilized to accelerate the healing process. The degree of neovascularization frequently reflects the progression of healing [18], where CD31 is a biomarker for emerging blood vessels, and α-SMA acts as a definitive marker for myofibroblasts, crucial in wound contraction and tissue fibrogenesis. Accordingly, skin wound tissues underwent immunostaining to detect CD31 and α-SMA. Illustrated in Figure 8A and B, the HA + 3D-Exos group exhibited a significant increase in CD31 levels by Day 7, denoting enhanced neovascularization post HA + 3D-Exos application. Figure 8D and E depict a marked rise in α-SMA, indicating an earlier transition into the proliferation phase for the HA + 3D-Exos group compared to the others.

Figure 8C and F show that by Day 14, both markers’ expressions significantly diminished in the HA + 3D-Exos and HA + 2.5D-Exos groups, while there was a substantial increase in the HA + 2D-Exos, HA, and CON groups. This implies that the HA + 3D-Exos and HA + 2.5D-Exos groups had progressed beyond the proliferation stage, moving into the remodeling phase. In contrast, the HA and CON groups were just starting to show evident new blood vessels, remaining in the proliferation stage with lagging tissue repair. The diminished levels of both markers in the HA + 3D-Exos group, in comparison to the HA + 2D-Exos, HA, and CON groups, suggest superior healing efficacy. The increased number of vessels in the HA + 3D-Exos group could be attributed to the amplified secretion of angiogenic factors by ADMSCs under a 3D coaxial biomimetic environment [45], likely enhancing neovascularization and maturation, thus effectively promoting wound healing.

## Discussion

Our study delineates a groundbreaking approach for burn wound healing that leverages the potential of ADMSC-derived exosomes, particularly those cultured in a 3D environment. The quintessential innovation lies in our high-performance exosome controllable releasing hydrogel system. This sophisticated design ingeniously combines 3D-cultured ADMSC-derived exosomes with a biocompatible HA matrix, thereby achieving a slow and controlled release of therapeutic exosomes directly into the wound site.

The rationale for adopting this composite system emerges from the inherent limitations plaguing conventional exosome delivery methods [13, 59], particularly low yield [27, 28] and rapid degradation. In contrast, 3D-Exos not only ameliorate these challenges but also manifest enhanced bioactivity, as demonstrated through *in vitro* and *in vivo* assays on parameters such as cell proliferation, migration, and angiogenesis. Moreover, the use of HA as a vehicle serves dual purposes: it offers a protective microenvironment for the exosomes and further augments their bioactivity by virtue of HA’s known biocompatible and biodegradable properties [60, 61].

Exosomes have long been celebrated for their proficiency in intercellular communication [13, 62, 63], yet their role in burn wound healing has largely remained unexplored. Our study stands at the forefront, empirically demonstrating the merits of exosomes cultured in a 3D environment, encapsulated within a HA hydrogel, in enhancing burn wound healing. The 3D architecture of our culture system not only boosts exosome production but also augments levels of pluripotency markers, thereby elevating their therapeutic potential.

Our research unveils a consistent application of chemically defined media and UC techniques across a spectrum of culture methods, including conventional 2D plate culture (2D-Exos), microcarrier culture (2.5D-Exos), and 3D-printed microfiber culture (3D-Exos). Distinct differences lie in the unique preparatory methods and the volume of medium employed [26, 62–64]. Notably, 2D culture requires a substantially larger volume of supernatant for processing, unlike microcarrier and 3D cultures. Yet, 3D-Exos, utilizing the least medium volume, exhibited superior cell enhancement effects, indicating more efficient resource utilization. This subtly emphasizes the benefits of the 3D-printed microfiber culture technique. Furthermore, variations in supernatant fluid volumes and compositions may impact exosome functionality and efficacy. Future investigations could delve deeper into how these differences in supernatant volumes affect exosome preparation outcomes.

In our exploratory studies on exosome preparation methodologies, we identified significant advantages of 3D and 2.5D culture systems over traditional methods. Exosome production is dichotomized into cell culture (upstream) and isolation (downstream) phases. The microcarrier culture (2.5D-Exos) excels in the cell culture phase, promoting substantial cellular proliferation at initial stages [32, 65]. This not only enhances cell yield but also influences cellular morphology and mechanosensitivity. These environmental-driven changes, manifesting in cytoskeletal and nuclear disposition variations, consequently affect cytokine production rates and the expression of specific cellular markers [33, 42, 64], potentially elucidating the superiority of exosomes derived from microcarrier cultures (2.5D-Exos) over conventional 2D plate cultures (2D-Exos).

Contrastingly, the 3D-printed microfiber culture (3D-Exos) encapsulates about 10^8^ cells within microfibers in a single dish, yielding a high concentration of Exos in minimal medium volume, significantly optimizing the cell culture and UC processes [26]. A salient aspect of this technique is the self-assembly of ADMSC in 3D environments, advancing our understanding of *in vivo* EV communication. This biomimetic approach produces exosomes with a diverse protein profile, aligning with observations where 3D-Exos significantly bolstered cellular proliferation and migration more effectively than 2D and 2.5D counterparts. Moreover, 3D-Exos exhibited substantial angiogenic effects in tube formation assays. However, the association between the protein diversity in 3D-prepared exosomes and their effectiveness in burn wound healing warrants further exploration [66, 67]. Beyond bioactive proteins, nucleic acid components such as miRNAs, circRNAs, lncRNAs, and piRNAs are speculated to considerably contribute to the reparative effects of 3D-Exos on skin tissue. Future research, employing advanced sequencing technologies, aims to decipher the specific molecular mechanisms underlying the efficacy of 3D-Exos. While the 3D-printed microfiber culture (3D-Exos) offers numerous benefits, its slower initial cell expansion phase remains a notable limitation. Future efforts may investigate the synergistic integration of microcarrier culture and 3D-printed microfiber culture, aiming to optimize and enhance the process across various dimensions, from stem cell extraction to a comprehensive, multi-dimensional analysis encompassing culture techniques, environmental conditions, separation methodologies, purification processes, storage strategies, and application standardization. Such advancements are expected to significantly accelerate the clinical translation of exosome-based therapies in tissue repair and regenerative medicine.

The multifaceted advantages of this system highlight its revolutionary potential in treating severe burns and suggest broader applications in tissue repair and regenerative medicine. Future research should further optimize exosome isolation and purification methodologies and examine interactions with the wound microenvironment throughout different healing phases to refine this promising therapeutic strategy.

Despite the progress, this study has limitations. Our investigation focused on the impact of exosomes on keratinocytes and endothelial cells, prompting further research into their effects on a wider array of cell types and stages of wound healing. Additionally, while our primary focus was on culture systems, there is room for a comprehensive survey of available isolation and purification methodologies [27, 44]. The next logical step involves developing an optimized, evidence-based protocol for exosome extraction from stem cells, incorporating a multi-dimensional analysis spanning culture techniques, conditions, separation methodologies, purification workflows, storage strategies, and standardization of use. Such advancements are expected to catalyze the translation of exosome-based therapies in tissue repair and regenerative medicine.

## Conclusion

In conclusion, our results delineate the engineering of a high-performance, exosome-controlled release hydrogel system designed explicitly for burn wound healing. Our unique system incorporates exosomes derived from 3D-printed cultures, offering not only a reduction in time and labor costs compared to conventional methods but also an enhancement in the biological functionality of the exosomes. Within this architecture, we have optimally integrated the advantages of 3D-cultured exosomes and HA loading, enabling the scalable, efficient, and high-quality preparation of exosomes, while concurrently enhancing their utility and long-term applicability. Our findings substantiate a framework for the future streamlined utilization of high-quality and high-yield 3D-cultured exosomes, as well as introduce a pioneering approach for the treatment of severe burns. We anticipate that such systems hold considerable promise for widespread applications in tissue repair and regenerative medicine in the near future.

## Supplementary Material

rbae035_Supplementary_Data
