# Supplementary material for: Enhanced burn wound healing by controlled-release 3D ADMSC-derived exosome-loaded hyaluronan hydrogel
Source: Regen Biomater. 2024 Mar 26;11:rbae035. doi: 10.1093/rb/rbae035 (PMC11018541; doi:10.1093/rb/rbae035)
Supplement: rbae035_Supplementary_Data [file rbae035_supplementary_data.docx]

Enhanced Burn Wound Healing by Controlled-Release 3D ADMSC-Derived Exosome Loaded Hyaluronan Hydrogel

Delong Zhu^#a^, Ying Hu^#a^, Xiangkai Kong^#a^, Yuansen Luo^c^, Yi Zhang^b^, Yu Wu^a^, Jiameng Tan^a^, Jianwei Chen*^d^, Tao Xu*^de^ and Lei Zhu*^a^

1. Department of Dermatology & Plastic Surgery, The Third Affiliated Hospital of Sun Yat-sen University, No. 600 Tianhe Road, Tianhe District, Guangzhou 510630, China.
2. Department of Research and Development, Huaqing Zhimei (Shenzhen) Biotechnology Co., Ltd., Shenzhen 518107, People’s Republic of China
3. Department of The Second Plastic Surgery, The First People’s Hospital of Foshan, Foshan 528000, China.
4. Center for Bio-Intelligent Manufacturing and Living Matter Bioprinting, Research Institute of Tsinghua University in Shenzhen, Tsinghua University, Shenzhen, 518057, People’s Republic of China.
5. Tsinghua Shenzhen International Graduate School, Tsinghua University, Shenzhen 518055, People’s Republic of China.

^#^These authors contributed equally to this work.

*Corresponding author. E-mail: chenjw@tsinghua-sz.org(J.C.) ;drtaoxu@yeah.net/xut@tsinghua-sz.org(T.X.); zhulei@mail.sysu.edu.cn (L.Z.)

**Supplementary info.**


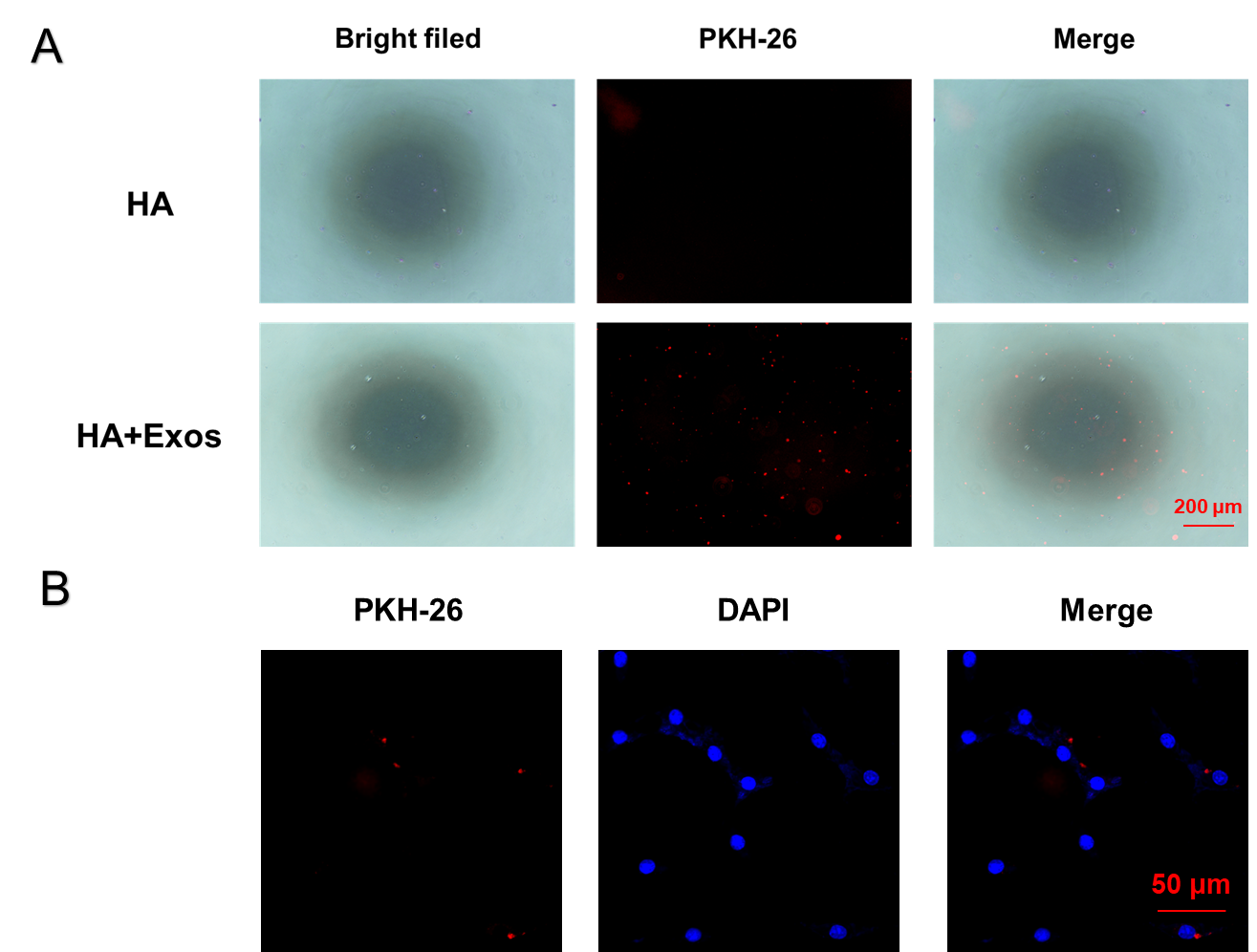


**Figure S1**. **Internalization of ADMSC -Exo.** (A) Fluorescence microscopy reveals PKH26-labeled ADMSC-Exos integrated into hyaluronic acid, showcasing the exosomes' distribution (Scale bar: 200µm). (B) Confocal microscopy images of HUVECs post a 24-hour incubation with PKH26-labeled HA-Exos (Scale bar: 50µm), illustrating the effective release of HA-Exos and their rapid internalization by HUVECs.

**1.****Internalization of** **ADMSC -Exo**

To further corroborate the effective incorporation of exosomes into the hyaluronic acid matrix, we utilized ADMSC-derived exosomes, labeled with PKH26 red fluorescent dye, and integrated them into the hyaluronic acid. Intriguingly, our imaging techniques unveiled a uniform distribution of ADMSC-Exos within the hyaluronic acid matrix, as detailed in Supplementary Figure S1A. The subsequent investigations, depicted in Figure S1B, entailed introducing these PKH26-labeled HA-Exos into the serum-free culture medium of HUVECs. Remarkably, post a 24-hour incubation period, we observed significant accumulation of these labeled exosomes in the perinuclear area of the HUVECs. This observation is indicative of the successful release and efficient internalization of ADMSC-Exos by HUVECs.

**
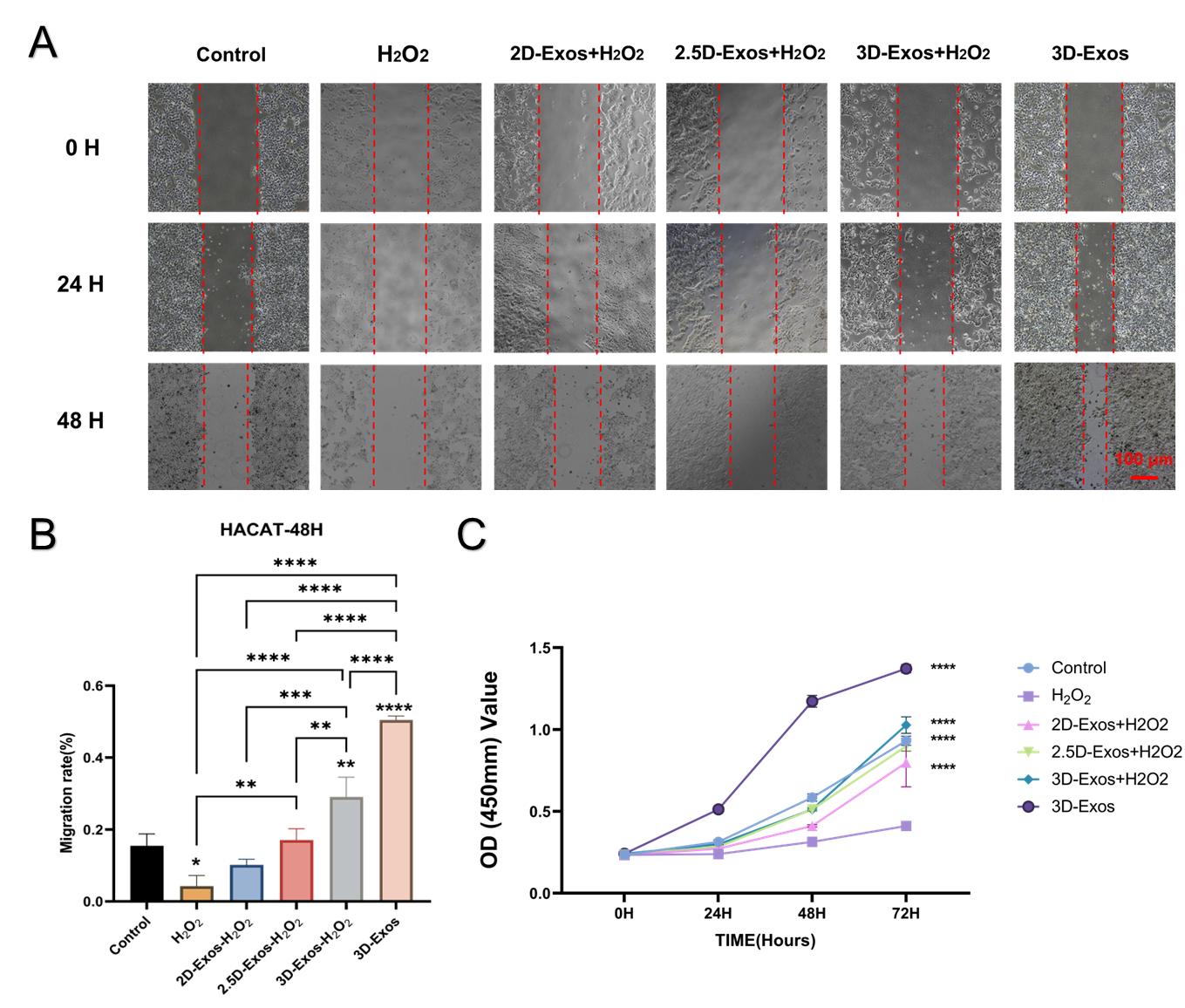
**

**Figure S2**. **Analysis of the Effects of ADMSC-Exosomes Derived from Various Preparation Techniques on HaCaT Cells in an H_2_O_2_ Milieu. (**A ,B) The migration response of HaCaT cells to different exosome formulations (3D-Exos, 3D-Exos+H_2_O_2_, 2.5D-Exos+H_2_O_2_, 2D-Exos+H_2_O_2_, H_2_O_2_, and Control) was evaluated using the scratch wound healing assay (Scale bar: 100µm). (C) The proliferation capacity of these cells under the corresponding treatments was assessed using a CCK-8 assay. Error bars denote standard deviations from triplicate experiments (*p < 0.05; **p < 0.01; ***p < 0.001; ****p < 0.0001).

**2.Analysis of the Effects of ADMSC-Exosomes Derived from Various Preparation Techniques on HaCaT Cells in an H_2_O_2_ Milieu.**

In the event of burn injuries, the impacted site is subjected to a high reactive oxygen stress microenvironment. To address this, our study has been extended to include a new experimental group. This group focuses on applying ADMSC-Exosomes, synthesized via multiple techniques, to HaCaT cells treated with H_2_O_2_, thereby mimicking the oxidative stress environment typical of burn wounds. This enhancement of our research protocol is designed to deepen our understanding of the impact of ADMSC-Exosomes, prepared under varying conditions, in high oxidative stress settings. Such an investigation is crucial for advancing our knowledge of the therapeutic potential of ADMSC-Exosomes in the context of burn wound healing under oxidative stress.

The scratch wound healing assay results indicated an enhanced migratory capability of HaCaT cells cultured with ADSC-Exosomes, irrespective of the presence or absence of H_2_O_2_. However, the effect of 3D-Exos on the migration of HaCaT cells, which was impaired by H_2_O_2_, was particularly notable (as shown in Figure S2A, B; *P < 0.05, **P < 0.01, ***P < 0.001). In Figure S2(C), we observed an increased optical density (OD) at 450 nm in the 3D-Exos group compared to the control group. This enhancement was also evident in the 3D-Exos+ H_2_O_2_, 2.5D-Exos+ H_2_O_2_, and 2D-Exos+ H_2_O_2_ groups in comparison to the H_2_O_2_ group. Notably, 3D-Exos significantly improved the proliferation of HaCaT cells that were impaired by H_2_O_2_.

These findings collectively suggest that ADMSC-Exosomes from different preparation methods facilitate cell proliferation and migration, with the 3D-Exos+ H_2_O_2_ group showing the most pronounced effect. This highlights the potential of 3D-Exos as a significant factor in skin wound healing, particularly in environments characterized by high reactive oxygen stress. This study provides valuable insights into the regenerative capabilities of ADMSC-Exosomes under oxidative stress conditions, reinforcing their therapeutic potential in wound healing applications.

**
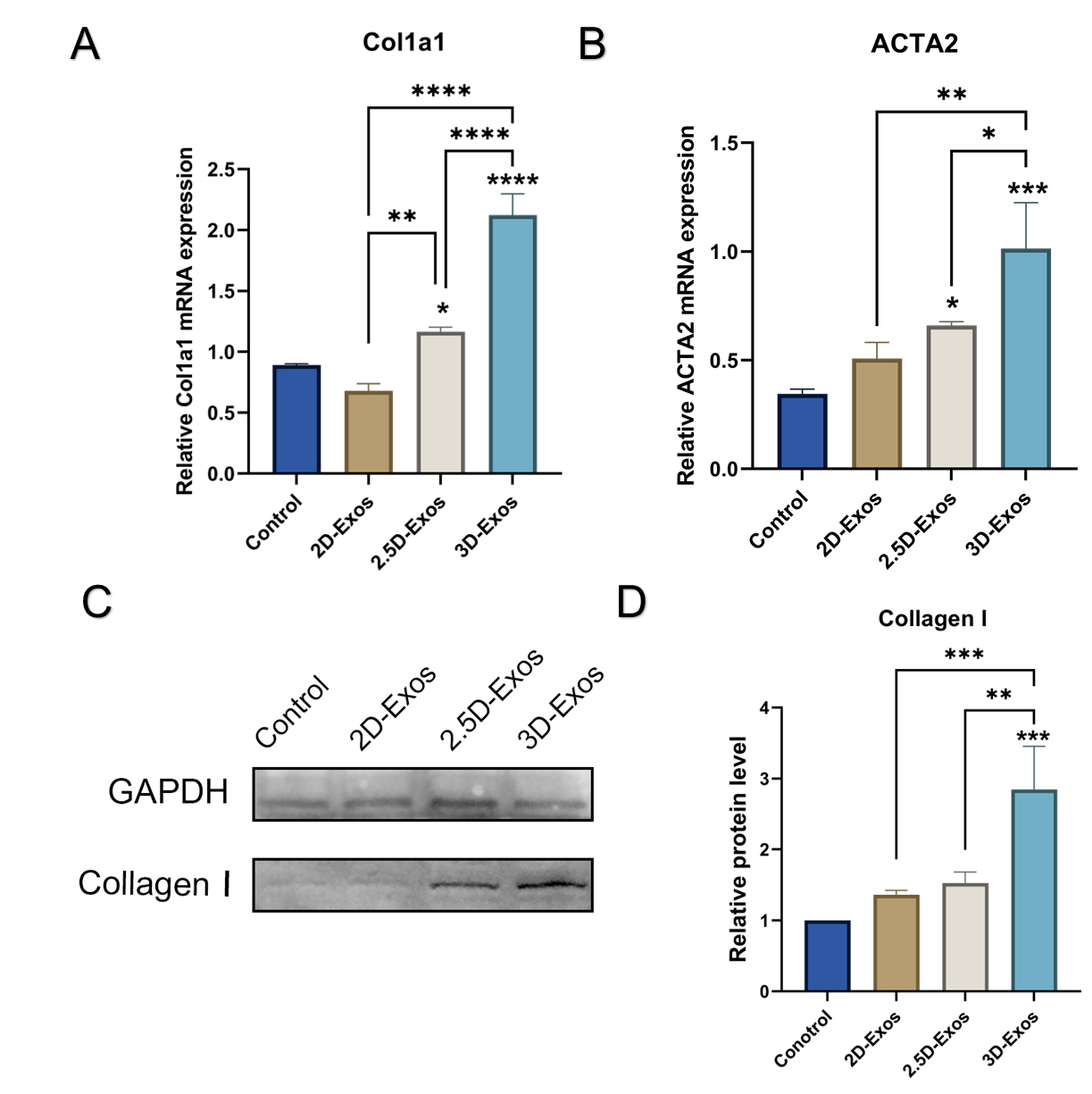
**

**Figure S3**. **The Influence of ADMSC-Exosomes Prepared by Different Methods on the Expression of Wound Healing-Related Genes in Dermal Fibroblasts.** (A ,B) mRNA expression of COL1A1, ACTA2 in human fibroblasts treated with control, 2D-Exos, 2.5D-Exos, and 3D-Exos for 24 hours. (C) Western blot analysis of the expression of Collagen I in fibroblasts treated with different ADMSC-exosomes for 24 hours. (D) Densitometric analysis of the western blot bands. Results are presented as mean ±SD; n =3; *p <0.05, **p <0.01, ***p < 0.001, compared with the control groups.

**3.The Influence of ADMSC-Exosomes Prepared by Different Methods on the Expression of Wound Healing-Related Genes in Dermal Fibroblasts.**

To explore the impact of ADMSC-exosomes, prepared via varied methods, on gene expression related to wound healing in dermal fibroblasts, we conducted a series of experiments. Dermal fibroblasts, key players in scar formation and wound healing, regulate both the synthesis and degradation of extracellular matrix (ECM) and collagen. It is well-documented that ADMSC-Exos can be internalized by these fibroblasts. In this study, we examined how ADMSC-Exos influence the expression of ECM-associated genes. As shown in Fig. S3(A,B), treatment with different concentrations of ADMSC-exosomes led to an upregulation in the mRNA expression of ACTA2 and Col1A1. Correspondingly, Fig. S3(C) reveals a similar increase in the protein levels of COL1A1 following exosome treatment. These results indicate that ADMSC-Exos, particularly the 3D-Exos formulation, not only elevate the ratio of α-SMA and Col1A1 but also align with our in vivo findings, reaffirming their significant role in enhancing skin wound healing.
